# Fluconazole induces ROS in *Cryptococcus neoformans* and contributes to DNA damage *in vitro*

**DOI:** 10.1371/journal.pone.0208471

**Published:** 2018-12-07

**Authors:** Congyue Annie Peng, Andrea A. E. Gaertner, Sarah Ana Henriquez, Diana Fang, Rodney J. Colon-Reyes, Julia L. Brumaghim, Lukasz Kozubowski

**Affiliations:** 1 Department of Genetics and Biochemistry, Clemson University, Clemson, South Carolina, United States of America; 2 Department of Chemistry, Clemson University, Clemson, South Carolina, United States of America; Yonsei University, REPUBLIC OF KOREA

## Abstract

Pathogenic basidiomycetous yeast, *Cryptococcus neoformans*, causes fatal meningitis in immunocompromised individuals. Fluconazole (FLC) is a fungistatic drug commonly administered to treat cryptococcosis. Unfortunately, FLC-resistant strains characterized by various degree of chromosomal instability were isolated from clinical patients. Importantly, the underlying mechanisms that lead to chromosomal instability in FLC-treated *C*. *neoformans* remain elusive. Previous studies in fungal and mammalian cells link chromosomal instability to the reactive oxygen species (ROS). This study provides the evidence that exposure of *C*. *neoformans* to FLC induces accumulation of intracellular ROS, which correlates with plasma membrane damage. FLC caused transcription changes of oxidative stress related genes encoding superoxide dismutase (*SOD1*), catalase (*CAT3*), and thioredoxin reductase (*TRR1*). Strikingly, FLC contributed to an increase of the DNA damage *in vitro*, when complexed with iron or copper in the presence of hydrogen peroxide. Strains with isogenic deletion of copper response protein metallothionein were more susceptible to FLC. Addition of ascorbic acid (AA), an anti-oxidant at 10 mM, reduced the inhibitory effects of FLC. Consistent with potential effects of FLC on DNA integrity and chromosomal segregation, FLC treatment led to elevated transcription of *RAD54* and repression of cohesin-encoding gene *SCC1*. We propose that FLC forms complexes with metals and contributes to elevated ROS, which may lead to chromosomal instability in *C*. *neoformans*.

## Introduction

*Cryptococcus neoformans* is a basidiomycetous yeast that causes pneumonia and meningitis primarily in immunocompromised patients, rendering approximately 620,000 deaths per year [1–3]. Global prevalence of cryptococcal disease is 5–10% in Americas and Europe, and exceeds 15% in South East Asia and Sub-Saharan Africa [4, 5]. Fluconazole (FLC), a well-tolerated triazole drug that accumulates in the cerebrospinal fluid is often chosen to initiate treatment of cryptococcal meningitis or as a maintenance therapy [6, 7]. However, due to the fungistatic effect of FLC, the emergence of resistance to the drug complicates treatments [8–10]. Clearly there is a need to develop more effective therapies and towards this goal demarcate mechanisms for drug resistance.

FLC has been used as an antifungal agent since 1990 [7, 11]. FLC inhibits lanosterol 14α-demethylase (Erg11), a conserved enzyme that catalyzes the reaction of converting lanosterol to ergosterol [12]. Fungal growth arrest upon exposure to FLC is attributed to the reduction of ergosterol in the plasma membrane combined with an accumulation of potentially toxic sterols [13]. The mechanisms that attribute to the increasing *C*. *neoformans* azole drug resistance include *ERG11* gene mutation [14], gene duplication [15], and drug efflux pump Afr1 [16,17]. In addition, a number of kinases that are involved in TOR signaling (Ypk1, Ipk1, Gsk3), related to vacuole transport (Vps15) and involved in cell cycle (Cdc7) are associated with FLC resistance [18].

Depletion of ergosterol has been associated with the disruption of V-ATPase function [19]. Only a single copy of the *ERG11* gene was mapped to *C*. *neoformans* chromosome #1 (Chr1) [12]. However, in FLC resistant strains, Chr1 was found to possess disomic duplication [15]. Integrity of endoplasmic reticulum is important for disomy of Chr1 and Chr4, leading to FLC resistance [20, 21]. Recent findings suggest that decoupling of cellular growth and nuclear division during FLC treatment leads to increased DNA content, which may be a conserved way to acquire azole resistance in fungal pathogens [22, 23]. However, the exact mechanisms underlying chromosomal changes in cells treated with FLC remain unknown.

Chromosomal instability has been associated with chronic oxidative stress mediated by elevated reactive oxygen species (ROS) and it is well established that ROS can lead to DNA damage. For example, human-hamster hybrid GM10115 cells acquired 22% chromosomal instability after exposure to H_2_O_2_ [24]. In the model organism *Saccharomyces cerevisiae* mutant strains with impaired DNA repair and reduced ROS scavenger proteins showed increased frequency of chromosomal rearrangement in H_2_O_2_ [25].

ROS, including hydroxyl radicals can be generated through the oxidation and reduction of metals *in vivo* and *in vitro*, according to the following reaction [26].

$$Fe(II)or\;Cu(I)+H_{2}O_{2}\to Fe(III)or\;Cu(II)+OH^{-}+∙OH$$

Metallothioneins (MTs) are cysteine-rich proteins acting as metal chelators to maintain physiological ROS concentrations [27,28]. Two copper-detoxifying MTs were identified in *C*. *neoformans* proteome: CMT1 (13.4 kDa) and CMT2 (20.1 kDa), both of which are upregulated by copper [29]. Metal chelating domains in *C*. *neoformans* MTs provide high capacity MT-Cu^I^ binding, which is critical for counteracting the first line of copper-based immunity of the host [30,31]. Impaired MT proteins resulted in ROS accumulation and cell cycle arrest in mice embryonic fibroblast cells [32]. Thus, accumulated evidence suggests that FLC leads to an increase of ROS in *C*. *neoformans*, and such response may lead to chromosomal instability. However, no direct study has addressed this possibility.

This study examines the effects of FLC on ROS in *C*. *neoformans*, and explores the effects of FLC on DNA integrity *in vitro*. The data presented here suggest that FLC treatment leads to increase of ROS concurrently with plasma membrane damage. FLC also triggers adverse transcription of genes encoding the primary antioxidant defense gene, the copper/zinc superoxide dismutase (*SOD1*) [33] and catalase (*CAT3*). Furthermore, FLC in complex with a redox-active transition metal enhances DNA damage *in vitro*. Consistent with FLC affecting DNA integrity, FLC treatment leads to transcription changes of genes associated with DNA repair and chromosome segregation (*RAD54* and *SCC1*). These findings suggest that FLC treatment results in increase of ROS in *C*. *neoformans* and this effect may lead to chromosomal instability.

## Results

### Fluconazole treatment leads to accumulation of ROS, which correlates with compromised membrane integrity

**Fig 1:**
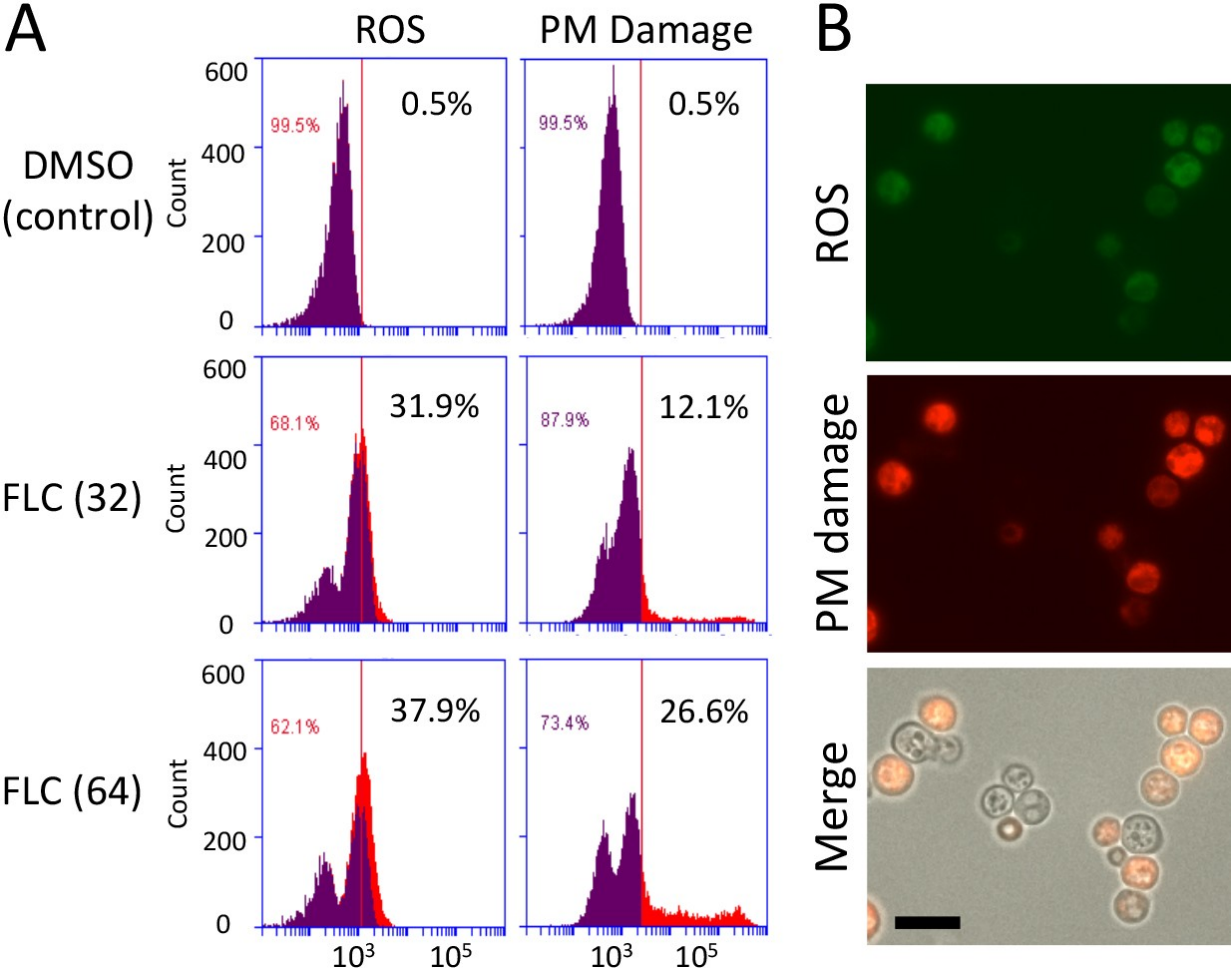
The effect of FLC treatment on ROS and plasma membrane integrity in *C*. *neoformans*. Cells (strain H99) were incubated in YPD medium supplemented with 0.1% (v/v) DMSO, 32 μg/ml (A) or 64 μg/ml (A, B) at 24°C for 8 (A) or 24 (B) hours. ROS (fluorescence of the H_2_DCFDA) and plasma membrane (PM) damage (fluorescence of the propidium iodide (PI)) was detected by either flow cytometry (A) or under fluorescence microscope (B). Vertical lines in the graphs indicate arbitrary boundary between background and elevated levels of the H_2_DCFDA and PI fluorescence whereas percentages indicate fraction of cells with elevated fluorescence. In the ROS graphs, purple area indicates the ROS content in the cell subpopulation that shows background PI fluorescence. Orange indicates ROS content in the cell subpopulation with elevated PI stain. Bar in B corresponds to 10 microns.

We hypothesized that FLC treatment leads to generation of ROS in *C*. *neoformans*. To test this possibility, we performed flow cytometry to simultaneously monitor intracellular ROS and plasma membrane integrity in *C*. *neoformans* treated with FLC. After 5 or 8 hours of treatment at 24°C with 32 μg/ml FLC, which is a concentration established as a heteroresistance level for the strain we have utilized in our studies (H99) [34], ROS accumulation was detected by the fluorescence shift of the ROS indicator H_2_DCFDA (Fig 1A and Figure A in S1 File). The effect of FLC on membrane integrity was concurrently monitored based on the intracellular incorporation of the propidium iodide (PI), a fluorescent dye that penetrates damaged plasma membrane [35] (Fig 1 and Figure A in S1 File). Percentage of cells with elevated fluorescence of H_2_DCFDA and PI was higher when the concentration of FLC was increased to 64 μg/ml indicating a dose-dependent response (Fig 1A). Flow cytometry data analysis revealed a positive correlation between elevated ROS and the increased PI fluorescence. This finding was further confirmed by fluorescence microscopy (Fig 1B). Exposure to FLC for 1 hour had no significant effect on ROS or plasma membrane integrity (Figure A in S1 File). To test if the observed effects of FLC were common to other *C*. *neoformans* strains or related species (beyond serotype A, var. *grubii*, represented here by strain H99), we included in our analysis two strains of *C*. *neoformans* var. *neoformans* (*C*. *deneoformans*, serotype D) and a strain of the sister species *Cryptococcus deuterogattii*. In all three cases, FLC led to a dose-dependent increase in ROS and concurrent plasma membrane damage (Figure B in S1 File). These data suggest that FLC treatment concurrently leads to increase of ROS and plasma membrane damage in *Cryptococcus*.

### Analysis of transcription of ROS-related genes in fluconazole-treated *C*. *neoformans*

**Fig 2:**
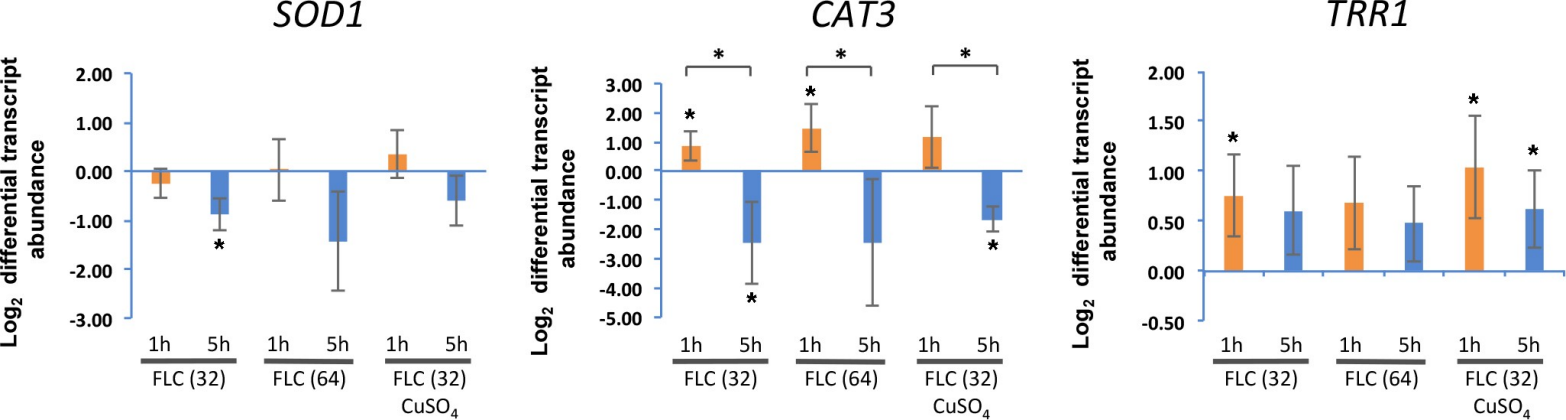
The effects of FLC on transcription of genes encoding copper/zinc superoxide dismutase (*SOD1*), catalase (*CAT3*) and thioredoxin reductase (*TRR1*). Exponentially grown cultures of *C*. *neoformans* (H99) were resuspended in YPD medium supplemented with FLC (32 or 64 μg/ml), CuSO_4_ (1 mM) with FLC (32 μg/ml), or 0.1% (v/v) DMSO (as control) and grown at 24°C for 1 or 5 hours prior to total RNA extraction. Transcript abundance was analyzed by quantitative PCR. Star indicates statistical significance (p < 0.05).

To further test possible effects of FLC on ROS, we examined whether FLC treatment affects transcription of genes encoding proteins involved in ROS reduction. We performed quantitative PCR to monitor transcript abundance of the following genes: copper/zinc superoxide dismutase (*SOD1*, CNAG_01019), catalase (*CAT3*, CNAG_00575) [36], thioredoxin reductase (*TRR1*, Accession # AY796185) [37], peroxiredoxin (CNAG_03482) [38] and *CMT1* and *CMT2*, two genes encoding metallothioneins (MTs). *C*. *neoformans* (strain H99) cells were treated for 1 or 5 hours with either 32 or 64 μg/ml FLC. Treatment with CuSO_4_ and 32 μg/ml FLC for 1 and 5 hours were also performed, as the transcription of *CMT1/2* was expected to increase in the presence of copper [29]. *SOD1* was modestly downregulated after 5 h of FLC incubation (log_2_ fold change = -0.89 ± 0.34 for 32 μg/ml FLC, *p* = 0.0103) (Fig 2). *CAT3* was upregulated after 1 h incubation at both FLC concentrations (log_2_ fold change = 0.86 ± 0.5, *p* = 0.0404; log_2_ fold change = 1.45±0.81, *p* = 0.0356 for 32 or 64 μg/ml FLC, respectively). However, *CAT3* was downregulated after 5 h of incubation with 32 μg/ml FLC (log_2_ fold change = -2.48 ± 1.45, *p* = 0.0383). The decrease of *CAT3* transcripts from 1h to 5h at both FLC concentrations were significant (*p* = 0.0181 and *p* = 0.0448 for 32 and 64 μg/ml FLC, respectively). In contrast FLC did not cause downregulation of *TRR1* and the transcription was upregulated at 1 h incubation at FLC 32 μg/ml (log_2_ fold change = 0.75 ± 0.41, *p* = 0.0341) (Fig 2). *CMT1* and *CMT2*, as predicted, exhibited elevated transcripts in the presence of CuSO_4_ (*CMT1* log_2_ fold change = 1.72 ± 0.41, *p* = 0.002; log_2_ fold change = 2.53 ± 0.41, *p* = 0.0004 for 32 or 64 μg/ml FLC, respectively; *CMT2* log_2_ fold change = 3.19 ± 0.61, *p* = 0.001; log_2_ fold change = 2.59 ± 0.33, *p* = 0.0002 for 32 or 64 μg/ml FLC, respectively) (Figure C in S1 File). However, the effect of FLC on *CMT1* and *CMT2* transcription was negligible (Figure C in S1 File). Additional treatment with CuSO_4_ did not potentiate the effects of FLC on transcription of *SOD1*, *CAT3*, and *TRR1* (Fig 2). Similar to *CMT*s, the gene encoding peroxiredoxin, found upregulated by oxidative stress in other studies, did not show a clear trend on gene regulation by FLC [38] (Figure C in S1 File). These data suggest that FLC treatment results in elevated expression of *TRR1* and the exposure to FLC for 5 hours leads to reduced expression of *SOD1* and *CAT3*. The result that these two genes were inhibited after 5-hour incubation correlates with flow cytometry data (Figure A in S1 File) that show ROS elevated after 5 but not 1 hour of incubation in media supplemented with FLC.

### Fluconazole coordinates to metal and changes metal redox potentials

Based on our results that suggest an increase of ROS in FLC-treated cells, we hypothesized that treatment with FLC leads to chromosomal instability through the mechanism that involves ROS. Previous studies have indicated that antimicrobial drugs can couple with metals and form metal complexes that potentiate DNA damage [39] Coordination of FLC to copper and iron in aqueous solution was confirmed using MALDI-TOF mass spectrometry (Table B and Figure D in S1 File). For both Fe^II^ and Cu^II^, two equivalents of FLC bound per metal ion. Even when metal:FLC ratios were increased to 1:4 or 1:6, only complexes with 1:2 ratio of metal to FLC were observed (Table B and Figure D in S1 File). In NMR spectroscopy studies, Nagaj *et al*. [40] observed metal-FLC complexes with a variety of metal:FLC ratios, including dimeric species, dependent upon pH/pD. Since we only observed 1:2 metal:FLC complexes under our conditions, we used this metal:FLC ratio in subsequent electrochemical and DNA damage studies.

Cyclic voltammetry (CV) experiments were performed on FLC in the presence of metals to evaluate how FLC binding alters the redox activity of copper and iron. All experiments were performed in aqueous solutions at physiologically relevant pH with potassium nitrate as supporting electrolyte, and the electrochemical data are provided in Table C and Figures E and F in the S1 File. FLC does not exhibit electrochemical activity. Upon addition of FLC to copper, the Cu^I/0^ and Cu^II/I^ redox potentials shift to more positive potentials relative to CuSO_4_ (*E*_1/2_ values of 250 vs. 11 mV, respectively), indicating that FLC binding stabilizes hydroxyl-radical-generating Cu^I^ over Cu^II^. Similar stabilization of Fe^II^ over Fe^III^ is observed upon iron-FLC binding (Fe^III/II^ *E*_1/2_ values shift from 65 mV in FeSO_4_ to 98 mV for Fe-FLC). In both cases, the redox potentials of the FLC-metal complexes are well within the potential window for biological hydroxyl radical generation (-320 to 460 mV) [41].

### Fluconazole-metal binding causes DNA damage *in vitro*

**Fig 3:**
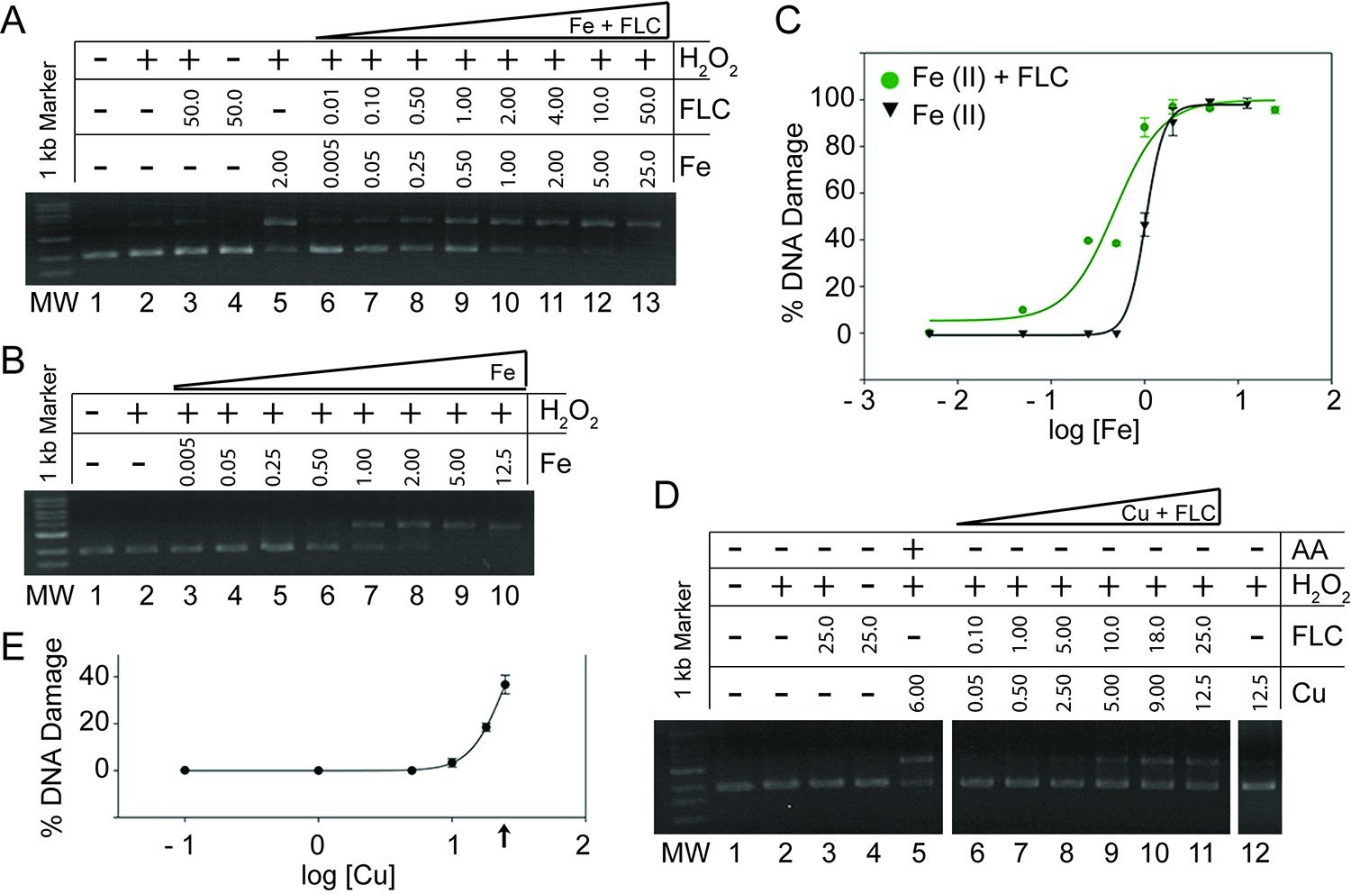
Fluconazole-metal complex causes DNA damage *in vitro*. A plasmid DNA (p) was incubated with reagents as indicated here and specified in the Materials and Methods. Samples were separated by electrophoresis, gels were stained with ethidium bromide and the amounts of nicked (damaged, upper band) and circular (undamaged, lower band) plasmid (p) were analyzed. In all gels shown (A, B and D), the MW: 1 kb molecular weight marker; lane 1: plasmid DNA (p); lane 2: p + H_2_O_2_. A) Fe^II^-mediated DNA damage stimulated by FLC. lane 3: p + FLC (50 μM) + H_2_O_2_; lane 4: p + FLC (50 μM); lane 5: p + FeSO_4_ (2 μM) + H_2_O_2_; lanes 6–13: p + H_2_O_2_ + increasing concentrations (μM) of FLC and FeSO_4_, as indicated. B) Fe^II^-mediated DNA damage. lane 3–10: p + H_2_O_2_ + increasing concentrations (μM) of FeSO_4,_ as indicated_._ C) Dose-response curves of iron-mediated DNA damage with and without addition of FLC, based on experiments described in A and B. D) Cu^II^-mediated DNA damage stimulated by FLC. lane 3: p + FLC (25 μM) + H_2_O_2_, lane 4: p + FLC (25 μM); lane 5: p + CuSO_4_ (6 μM) + AA (7.5 μM) + H_2_O_2_; lanes 6–11: p + H_2_O_2_ + increasing concentrations (μM) of FLC and CuSO_4_, as indicated; lane 12: p + CuSO_4_ (12.5 μM) + H_2_O_2_ E) Dose-response curve of copper-FLC-mediated DNA damage. An arrow indicates the highest concentration of CuSO_4_ at which there was no DNA damage detected when FLC was not included.

The ability of FLC to promote DNA damage in the presence of iron and copper was investigated using gel electrophoresis studies under physiologically relevant conditions. DNA damage caused by increasing concentrations of FLC and iron or copper (in a 2:1 ratio) is shown in Fig 3; complete data tables for these studies are provided in Tables D-F in the S1 File. For the purpose of this evaluation, we defined DNA damage as percentage of shift between the fluorescent band corresponding to intact (circular) DNA and the band corresponding to the nicked (damaged) DNA, as specified in more details in Materials and Methods. Lanes 2 in Fig 3A, 3B and 3D show that hydrogen peroxide alone does not cause DNA damage. Similarly, FLC in the presence or absence of hydrogen peroxide does not cause DNA damage (Fig 3A and 3D, lanes 3 and 4). Quantification of the signal in lane 5 (Fig 3A) shows that Fe^II^ (FeSO_4_, 2 μM) and hydrogen peroxide cause ~90% DNA damage. Increasing concentrations of FLC and Fe^II^ in the presence of hydrogen peroxide caused DNA damage in a dose-dependent manner (Fig 3A, lanes 6–13). Importantly, within the Fe^II^ concentration range of 0.005–1 μM, addition of FLC at the 2:1 ratio had a significant potentiating effect on DNA damage (Fig 3A, lanes 6–10 vs. Fig 3B, lanes 3–7, quantified and graphed in Fig 3C). Statistical analysis indicated that the effect of FLC was significant (Table G in S1 File). At Fe^II^ concentrations of 2 μM and above, FLC had no additional effect (Fig 3A, lanes 11–13 vs. Fig 3B, lanes 8–10, and Fig 3C).

FLC also exhibited a potentiating effect on DNA damage when complexed with copper. Cu^II^ in the presence of hydrogen peroxide did not cause DNA damage at concentrations of up to 12.5 μM (Fig 3D, lane 12). However, when Cu^II^ was reduced to Cu^I^ with an addition of stoichiometric amounts of AA, it caused DNA damage in the presence of hydrogen peroxide (Fig 3D, lane 5). Strikingly, even in the absence of AA, DNA damage was observed in the presence of hydrogen peroxide with increasing concentrations of FLC and Cu^II^ at concentrations of Cu^II^ ranging from 5 to 12.5 μM (Fig 3D, lanes 6–11, and Fig 3E). Thus, the observed DNA damage results from copper-FLC binding. A maximum of 40% DNA damage was observed with 25 μM FLC and 12.5 μM Cu^II^ (Fig 3D, lane 11). Since FLC by itself does not result in DNA damage, this highlights the importance of metal binding on FLC-mediated damage. These plasmid damage assay results indicate that FLC binding to iron and copper significantly increases the metal’s ability to generate ROS and damage the DNA.

### Fluconazole treatment leads to differential transcription of genes related to DNA repair

**Fig 4:**
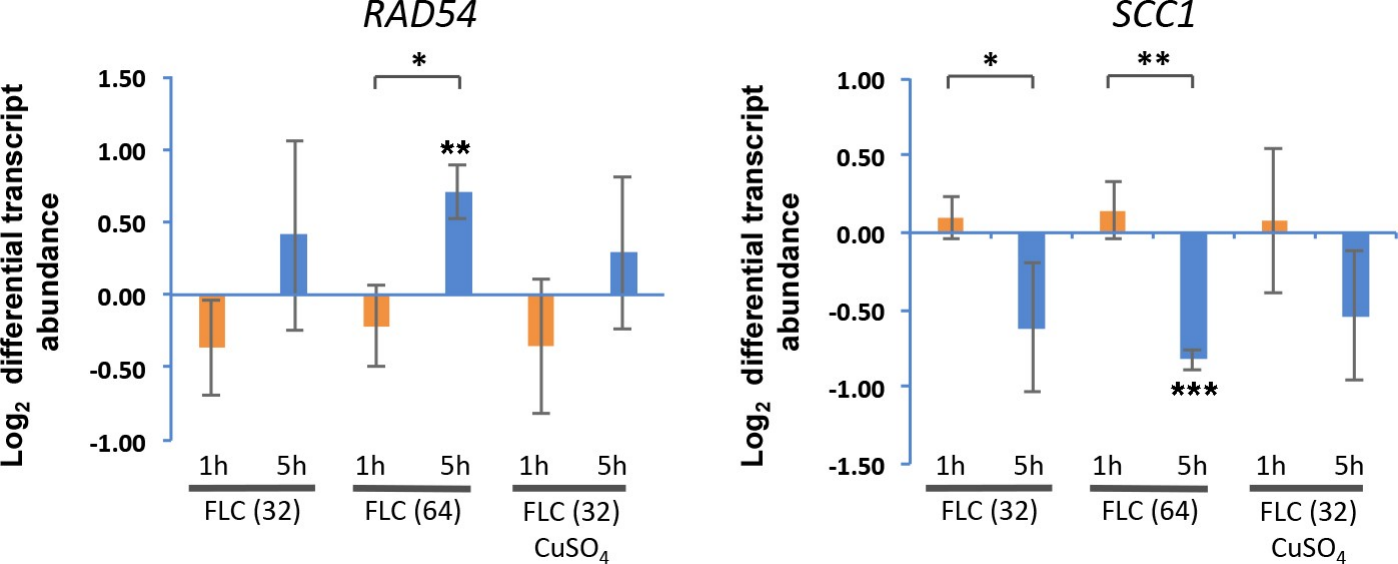
The effects of FLC on transcription of *C*. *neoformans* DNA repair genes (*RAD54*, and *SCC1*). Exponentially grown cultures of the *C*. *neoformans* (H99) were resuspended in YPD medium supplemented with FLC (32 or 64 μg/ml), CuSO_4_ (1 mM), or 0.1% (v/v) DMSO (as control) and grown at 24°C for 1 and 5 hours prior to total RNA extraction. Transcript abundance of DNA repair genes was analyzed using quantitative PCR. Statistical significance is indicated as single (p < 0.05), double (p < 0.01), or triple star (p < 0.001).

Our findings of FLC-mediated DNA damage *in vitro* prompted us to investigate effects of FLC on DNA integrity *in vivo*. To start addressing this possibility we examined the effect of FLC treatment on transcript abundance of genes that are implicated in DNA repair. *C*. *neoformans* var. *grubii* homologues of the following DNA repair-associated genes were tested: *RAD51* (CNAG_00072), *RDH54* (CNAG_02771), and *RAD54* (CNAG_01163) [42]. We also tested the homologue of *SCC1/MCD1* (CNAG_01023), which encodes a subunit of cohesin complex involved in DNA repair and shown to be downregulated upon treatment with hydrogen peroxide [43]. Cells were treated for 1 or 5 hours with either 32 or 64 μg/ml FLC prior to RNA isolation and the samples were subject to subsequent quantitative PCR analysis. Transcriptional levels of *RAD51* and *RDH54* were variable between samples and did not exhibit consistent change (Figure G in S1 File). *RAD54* transcription was significantly induced after 5 h incubation at FLC 64 μg/ml (log_2_ fold change = 0.71 ± 0.19, *p* = 0.0028) (Fig 4). The increase of *RAD54* transcripts from 1h to 5h at FLC 64 μg/ml was significant (*p* = 0.0087). *SCC1* was downregulated after 5 h incubation at FLC 64 μg/ml (log_2_ fold change = -0.82 ± 0.07, *p* = 0.0001). The decrease of *SCC1* transcripts from 1h to 5h at both FLC concentrations was significant (*p* = 0.047 and *p* = 0.001 for 32 and 64 μg/ml FLC, respectively). As our *in vitro* data suggest the effects of FLC on DNA integrity depend on coordination with metals, we tested if addition of CuSO_4_ to the media would show a synergistic effect with FLC on expression of DNA repair-related genes. Contrary to this possibility, addition of CuSO_4_ did not lead to a significant effect on the expression of *RAD54* in samples incubated with FLC for 1 or 5 hours (Fig 4). For *SCC1*, the addition of CuSO_4_ to samples treated with FLC resulted in a non-significant difference in expression between 1 and 5 hours (Fig 4). In summary, these data suggest that FLC treatment for 5 hours leads to repression of *SCC1* and modest upregulation of *RAD54*.

### The ascorbic acid reduces inhibitory effects of fluconazole

Generation of ROS and the associated membrane damage upon exposure to FLC suggest that ROS contributes to growth inhibition elicited by FLC. If that was the case, simultaneous addition of an antioxidant should alleviate inhibitory effects of FLC. Furthermore, although MTs transcript abundance fluctuation was negligible with qPCR assay, MTs incorporate metals and counteract the inhibitory effects of ROS [27]. We tested the sensitivity to FLC of mutants lacking MTs and we also tested the effect of ascorbic acid (AA), which is known to possess antioxidant properties at 10 mM concentration towards *S*. *cerevisiae* [44].

**Fig 5:**
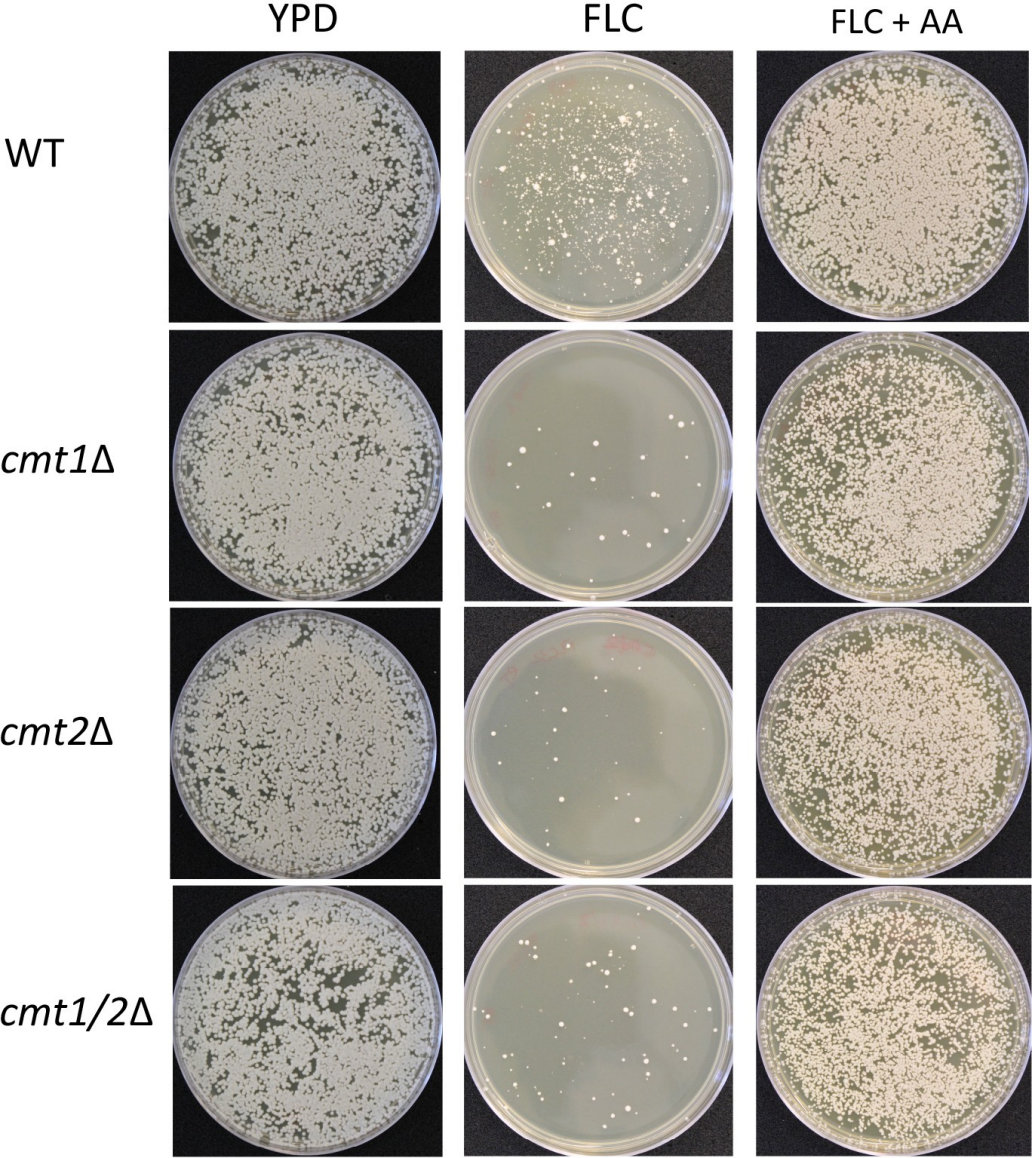
Anti-oxidant effects of ascorbic acid on *C*. *neoformans* H99 and *cmt1*Δ, *cmt2*Δ and *cmt1/2*Δ strains upon fluconazole treatment. Approximately 10,000 cells were plated on YPD and YPD supplemented with 32 μg/ml FLC with or without 10 mM ascorbic acid (AA).

Approximately 10,000 *C*. *neoformans* cells of the wild type (H99), and congenic mutants *cmt1*Δ, *cmt2*Δ, or *cmt1/2*Δ [29] were transferred to YPD plates supplemented with 32 μg/ml FLC, or a combination of 32 μg/ml FLC and 10 mM AA and the growth at 24°C was examined after 2 (for the controls) and 4 (for the FLC treatments) days. On the control plates (YPD), both the wild type and the three *CMT* mutants grew equally well (Fig 5). Previous findings show that exposure of wild type cells to 32 μg/ml FLC at 24°C results in heterogenous response with colonies of variable sizes [45]. Reassuringly, the wild type treated with FLC developed into colonies of variable sizes after 4 days at 24°C (Fig 5). In contrast, all three *CMT* mutants formed significantly fewer colonies at 24°C, suggesting that MTs are positively contributing to growth in the presence of FLC (Fig 5). Interestingly, the colonies of the *CMT* mutants that developed on FLC-containing media appeared less heterogeneous in size as compared to the wild type. Specifically, the population of mutant colonies appeared to lack the smaller colonies and consist mostly of larger colonies. Strikingly, addition of 10 mM AA significantly reduced the inhibitory effect of FLC towards the wild type and all three *CMT* mutants (Fig 5).

Given the positive effect of AA on growth in the presence of FLC, we expected lower levels of ROS in FLC-treated cells to which AA was added. Consistent with this possibility addition of AA to FLC-treated cells resulted in reduced levels of ROS and plasma membrane damage after 5 hours of incubation (Figure A in S1 File). However, at 8 hours the effect of AA was not detected (Figure A in S1 File). Percentage of *CMT* mutant cells with elevated H_2_DCFDA fluorescence was not significantly different from that of wild type at 1 and 8 hours (Figure H compared to Figure A in S1 File). However, at 5 hours of incubation with FLC, the *cmt2*Δ and *cmt1/2*Δ, and to a lesser extend the *cmt1*Δ, exhibited elevated ROS and the effect of AA was diminished as compared to that of the wild type (Figure H compared to Figure A in S1 File). Together our data suggest that the inhibitory effects of FLC are resulting, at least in part, from the accumulation of ROS. Moreover, MTs play an important role in *C*. *neoformans* ability to grow in the presence of FLC.

## Discussion

In this report, we demonstrate an increase of intracellular chronic oxidative stress (elevation of ROS) in FLC-treated *C*. *neoformans* and enhanced DNA damage by FLC in complex with redox active iron or copper *in vitro*. In addition, FLC treatment of *C*. *neoformans* led to transcriptional changes of genes involved in antioxidant responses and DNA repair *in vivo*. Together, these results implicate the role of FLC and the auxiliary metals in the generation of oxidative stress and chromosomal instability.

### Oxidative stress in fluconazole-treated *C*. *neoformans*

Previous studies have suggested that increase of ROS upon FLC treatment occurs also in other fungi, which supports our findings. Elevation of ROS has been reported in FLC-treated *Candida tropicalis* [46]. The same study showed that *C*. *tropicalis* strains resistant to FLC did not exhibit a spike of intracellular ROS when subsequently treated with FLC. We observed that *C*. *neoformans* cells from colonies that have developed on YPD medium supplemented with 32 ug/ml FLC exhibit heterogeneous response to subsequent FLC treatment with respect to ROS levels (Figure I in S1 File) reflecting heterogeneity of response to FLC at the population level [45]. Oxidative stress response markers, thioredoxin reductase and oxidoreductase, were induced by FLC in *Candida albicans* [47]. Consistent with these findings our study showed elevated levels of *TRR1* transcription in FLC treated cells. On the other hand two other genes involved in response to oxidative stress, *SOD1* and *CAT3* were downregulated after 5 hours of FLC exposure suggesting that increase in intracellular ROS may result from downregulation of *SOD1* and *CAT3*. *C*. *neoformans* accumulates SOD1 on the cytoplasmic membrane for a rapid anti-oxidant activity to cope with the external oxidative stress that is imposed by host defenses [48,49]. *SOD1* upregulation has been implicated in FLC-resistant *C*. *neoformans* strain, further suggesting that FLC may cause elevation of ROS through affecting *SOD1* expression [50].

Based on former studies [51], we suspected that FLC treatment leads to decreased expression of genes encoding metallothioneins (MTs). MTs play important roles in cell cycle regulation, apoptosis and controlling the levels of ROS via copper binding capacity and by donating electrons [28] [30]. A decrease in transcription of MT-encoding genes has been reported in FLC-treated *Microsporum canis* [51]. The importance of MTs in FLC resistance has been demonstrated in a study where FLC-resistant *Candida glabrata* exhibited significant upregulation of MT genes and increased levels of MT proteins [52, 53]. However, results of our qPCR experiments were inconsistent and did not show signifcant changes in levels of MT-encoding genes. On the other hand, mutant strains lacking MT activity developed less colonies on FLC-supplemented media suggesting the role of MTs in FLC-mediated growth inhibition.

Intracellular ROS accumulation in FLC-treated *C*. *neoformans* may contribute to the inhibition of growth, similar to ROS-induced growth arrest in *S*. *cerevisiae* [54]. Consistent with this possibility, we demonstrated here that 10 mM ascorbic acid (AA) decreased FLC potency against *C*. *neoformans*. In agreement with our data, an *in vivo* study of *C*. *albicans*-infected mice has suggested that AA decreased the effectiveness of FLC treatment [55]. Importantly, the same study demonstrated *in vitro* that addition of AA and two other antioxidants, N-acetylcysteine, and the reduced form of glutathione lead to improved growth of FLC-treated *C*. *albicans* [55]. Unexpectedly, our flow cytometry data showed that ROS levels were only reduced after 5 hours of incubation with FLC + AA but not 8 hours, although AA alleviated the effects of FLC on plasma membrane damage at both timepoints. One explanation of this finding could be that the antioxidant role of AA in *C*. *neoformans* is not based on direct ROS scavenging. It may alleviate the adverse effects of ROS rather than significantly reduce the amount of ROS. Furthermore, while AA has been shown to act as a scavenging sink for radicals, it can also convert to dehydroascorbate, where ascorbyl radicals are generated as intermediate products [56]. Although the ROS detection reagent H_2_DCFDA used here is presumably specific to ROS, we cannot preclude the possibility that H_2_DCFDA is oxidized by the intermediate products ascorbyl radicals, giving a false indication of ROS amount inside the cell. Alternatively, extensive culture aeration during incubation may simply lead to deactivation of the antioxidant potential of AA. On the other hand, the colony growth rescue by AA could also be due to its effects on ergosterol levels [57, 58]. Van Hauwenhuyse et al. (2014) hypothesize that AA effects are independent of its antioxidant potential based on the fact that addition of this compound did not result in change of ROS levels. The authors find that the effects of AA were dependent on the transcription factor Upc2, which regulates levels of ergosterol. It is still possible however that the effects of AA described by Van Hauwenhuyse et al. involved antioxidant activity that does not involve reduction of ROS (or the reduction was not detected due to reasons outlined above) [57].

### Effects of fluconazole on DNA integrity

DNA damaging effects of phenolic compounds mediated by iron and copper have been reported, supporting the idea that DNA damage caused by FLC *in vitro* is also mediated by coordinated metals [59–61]. Previous studies have demonstrated DNA damage *in vitro* by imidazole-based compounds complexed with metals [39, 62]. Chemotherapeutic agents may cause direct DNA damage through agent-DNA binding and indirect damage through inhibition of DNA repair or accumulation of intracellular ROS [63]. FLC causes diminishment of ergosterol and change in biophysical properties of cellular membranes and may also result in nuclear envelope leakage, which would facilitate FLC access to the DNA. Therefore, it is plausible that DNA damage in FLC-treated *C*. *neoformans* may be elevated and further potentiated by the accumulation of ROS. Consistent with FLC affecting DNA integrity, treatment with FLC led to upregulation of DNA repair gene *RAD54* and downregulation of *SCC1* homologue. Interestingly, it has been demonstrated that a 90-minute pre-treatment of *C*. *neoformans* with FLC leads to subsequent enhanced resistance to gamma radiation [42]. In light of our findings, it is possible that pre-treatment with FLC may prepare the cells for gamma radiation through differential regulation of DNA repair genes. Testing this hypothesis will require further investigations.

### The role of ROS and DNA damage in development of resistance to fluconazole

The evidence gathered in this study leads us to speculate that ROS is a double-edged sword in *C*. *neoformans* response to FLC. Accordingly, FLC would generate oxidative stress that impedes colony growth. On the other hand, similar to bacterial drug resistance developed through genetic variation triggered by antimicrobials [64], ROS and the available redox-active metals would induce DNA damage and chromosomal rearrangement that would increase a chance of developing FLC resistance. Elevated expression of *RAD54* upon FLC treatment reported here suggests possible mechanism of FLC-induced chromosomal translocations. This is supported by findings that show critical role of Rad54 in formation of bridge-induced translocation (BIT) in *S*. *cerevisiae* [65, 66]. Overexpression of *RAD54* resulted in an ensemble of secondary rearrangements between repeated DNA tracts after the initial translocation event, leading to severe aneuploidy with loss of genetic material [65]. Interestingly, *RAD54* is found over-expressed up to 5-fold in prostatic cancer cells characterized by recurrent non-reciprocal translocations [67]. Thus, FLC may affect chromosomal instability through a mechanism that is common to genomic rearrangements in cancer cells. Another intriguing finding in our study is the negative effect of FLC on the expression of *SCC1*, which encodes an important component of cohesin complex critical for the separation of sister chromatids [43]. As diminishment of cohesion between sister chromatids is associated with age-related increased incidence of aneuploidy [68], our data suggest possible mechanisms through which FLC treatment may increase a chance for chromosomal missegregation in *C*. *neoformans* which has been reported recently [22]. Further investigation is anticipated to test whether the elevated expression of *RAD54* and repression of *SCC1* upon FLC treatment contribute to DNA damage and chromosomal instability in *C*. *neoformans*.

The qPCR analysis performed in this study relies on total RNA. Therefore, the resulting data demonstrated the overall transcripts changes of the entire population at the time of RNA extraction. Such analysis does not reveal changes in individual cells. It needs to be emphasized that development of resistance to FLC may rely only on a small subset of cells. Considering the heterogeneity of the response to FLC, it is plausible that gene transcription levels are significantly variable between cells. This possibility is consistent with highly variable ROS levels in individual cells when *C*. *neoformans* is treated with FLC, as indicated by our flow cytometry analysis. Based on this reasoning we postulate that even modest changes in expression of genes relevant to oxidative stress and DNA repair that are observed at a population level may reflect a highly heterogeneous expression in individual cells. Furthermore, cells experiencing significant changes in gene expression upon FLC treatment would be critical for the development of resistance to FLC. It will be important to employ methods to study transcription activity in FLC-treated *C*. *neoformans* at a single cell level to further test this possibility.

## Materials and methods

### *Cryptococcus neoformans* strains

*Cryptococcus neoformans* var. *grubii* wild type (strain H99, Heitman Lab #4413), *C*. *neoformans* var. *neoformans* (*C*. *deneoformans*, strains JEC20, JEC21) and *C*. *deuterogattii* (strain R265, Heitman Lab #3030) were obtained from the strain collection of the laboratory of Dr. Joseph Heitman, Duke University. The *cmt1*Δ, *cmt2*Δ, *cmt1/2*Δ deletion mutants isogenic to H99 (*CMT1*, CNAG_05549; *CMT2*, CNAG_00306) were kindly provided by the laboratory of Dr. Dennis Thiele, Duke University [29].

### ROS and membrane permeability measurements

Cells were grown overnight at ambient temperature in 2 ml liquid YPD medium (1% yeast extract, 2% peptone, 2% dextrose) with constant agitation. Cells were centrifuged at 3000 x g for 2 min and resuspended in 850 μl YPD medium. 50 μl of the cell suspension was added into 2 ml YPD medium supplemented with either 0.1% (v/v) DMSO, or FLC at various concentrations as specified in figure legends. The approximate initial cell density for each treatment was 0.2 (OD_660_). The cells were incubated at ambient temperature with constant agitation for 1, 5, 8, 9, or 24 hours. At each time point, cells were harvested from the culture medium and centrifuged at 3000 x g for 2 min and washed with PBS (137 mM NaCl, 2.7 mM KCl, 10 mM Na_2_HPO_4_, 1.8 mM KH_2_PO_4_). To detect ROS, cells were resuspended in PBS supplemented with 20 μM 2’,7’-Dichlorofluorescin diacetate (H_2_DCFDA, Sigma Aldrich) and incubated in the dark at ambient temperature with constant agitation for 20 min. To detect membrane damage, cells were washed with PBS, resuspended in PBS supplemented with 5 μg/ml propidium iodide (PI, Sigma Aldrich, P417) and incubated in the dark at ambient temperature with constant agitation for 40 min. Cells were washed with PBS, resuspended in PBS, and sonicated at 20% output strength for 5 seconds. Fluorescence was detected by flow cytometry with the Accuri C6 instrument (BD Biosciences, San Jose, CA) based on FL1 533/30 and FL3 670 LP filter settings to detect ROS and the PI, respectively. Cells were visualized by Zeiss Axiovert 200 inverted microscope (Carl zeiss, Inc., Thornwood, NY) at 100x magnification.

### RNA extraction and qPCR

Cultures were grown in YPD liquid to exponential phase. The cells were harvested and resuspended in YPD medium each supplemented with either 32 or 64 μg/ml FLC, 32 μg/ml FLC and 1 mM CuSO_4_, or DMSO (as control) and grown at ambient temperature with constant agitation for 1 or 5 hours. RNA extraction was performed based on instruction of Bio-Rad Aurum Total RNA Mini Kit (Cat # 732–6820). Instead of using lyticase, the cells were mixed with 800 μl lysis buffer and 50 μl glass beads (Sigma G8772, 425–600 μm) and mechanically broken at 4°C with Minibeadbeater (3450 RPM, Biospec Products Model 607) for ten 20 second cycles with 2 min intervals.

First strand cDNA synthesis was performed per the instruction for the MnLV reverse transcriptase (New England Bio Lab M0253S). Parallel control reactions were set up that included all reagents except the MnLV reverse transcriptase. Reverse transcription was stopped by heating at 65°C for 20 min. The reaction was then diluted with water at 1:5. The amplification of each primer set was confirmed with end-point PCR. Primers utilized in this analysis are listed in Table A in S1 File.

qPCR was performed using Bio-Rad C1000 Touch Thermal Cycler with CFX96^TM^ Real-Time PCR System. iQ SYBR Green Supermix (BioRad #1708880) was used as fluorescent indicator. Forty PCR cycles were set at denaturation at 95°C for 10 seconds, annealing at 55°C for 30 seconds and primer extension at 72°C for 30 seconds. Primer efficiency was calculated from the standard curve generated from serial dilution of the control samples. The results were normalized based on the *C*. *neoformans* GAPDH gene (CNAG_06699). Melting curve was generated after each run to monitor the quality of the amplification products. Unpaired two samples t-test assumed equal variance was performed with R Studio version 3.4.4 and GraphPad Software.

### Fluconazole resistance plate assay

Approximately 10,000 cells in exponential growth in liquid YPD were spread onto plates containing various media and the colonies were inspected after 2 (for control) and 4 (for treatments) days of incubation at 24°C. To determine the influence of AA on FLC resistance, cells were spread onto YPD (control), or YPD plates supplemented with only FLC (32 μg/mL), or FLC and 10 mM AA.

### Fluconazole-metal binding mass spectrometry

Matrix-assisted laser desorption ionization-time of flight mass spectrometry (MALDI-TOF MS) experiments were performed using a Bruker Microflex mass spectrometer with trans-2-[3-(4-tert-butylphenyl)-2-methyl-2-propenyldiene (250.34 *m*/*z*) as the matrix. Samples with 1:1 and 1:4 Cu^II^: FLC ratios were prepared by combining aqueous solutions of CuSO_4_ (100 μL; 300 μM) and fluconazole (100 μL; 300 or 1200 μM, respectively). Samples with 1:1 and 1:6 Fe^II^: FLC ratios were made by combining aqueous solutions of FeSO_4_ (100 μL; 300 μM) and fluconazole (100 μL; 300 or 1800 μM, respectively).

### Electrochemical studies of FLC and FLC-metal complexes

Cyclic voltammetry (CV) were conducted with a CH Electrochemical Analyzer (CH Instruments, Inc.) at a sweep rate of 100 mV/s using a glassy carbon working electrode, a Pt counter electrode, and a Ag/AgCl (+0.197 V vs. NHE) [69]. All experiments were externally referenced to potassium ferricyanide (0.361 V vs. NHE) [70]. Studies were conducted in degassed MOPS buffer (10 mM, pH 7.0) for FLC and copper studies or MES buffer (10 mM, pH 6.0) for iron studies, with KNO_3_ (100 mM) as a supporting electrolyte. For iron binding studies, a solution of 1:2 iron-FLC ratio was made by adding aqueous solutions of FeSO_4_ (3 mL; 900 μM) and FLC (3 mL; 1800 μM), then diluting with the MES buffer (3 mL; 30 mM). For the copper study, a 1:2 solution was made by adding CuSO_4_ (3 mL; 300 μM) to FLC (3 mL; 900 μM), then diluting with the MOPS buffer (3 mL; 30 mM). All samples were deaerated for 10 minutes with N_2_ before each experiment. Samples were cycled between -0.6 and 1.0 V for copper and -1.0 and 1.0V for iron complexes.

### Plasmid DNA transfection, amplification, and purification

Plasmid DNA (pBSSK) was purified from DH1 *E*. *coli* competent cells (kindly provided by Dr. Stuart Linn’s lab, UC Berkeley) using a Zyppy^TM^ Plasmid Miniprep Kit (400 preps.). Plasmid was dialyzed against 130 mM NaCl for 24 hours at 4°C to ensure all Tris-EDTA buffer and metal contaminates were removed, and plasmid concentration was determined by UV-vis spectroscopy at a wavelength of 260 nm. Absorbance ratios of A250/A260 ≥ 0.95 and A260/A280 ≥ 1.8 were determined for DNA used in all experiments. Plasmid purity was determined through digestion of plasmid (0.1 pmol) with Sac1 and Kpn1 in a mixture of NEB buffer and bovine serum albumin at 37°C for 90 minutes. Digested plasmids were compared to an undigested plasmid sample and a 1 kb molecular weight marker using gel electrophoresis.

### DNA damage evaluation by gel electrophoresis

Deionized water, MOPS buffer (10 mM, pH 7.0), NaCl (130 mM), ethanol (100% metal free, 10 mM), as well as the indicated concentrations of CuSO_4_∙5H_2_O, AA (7.5 μM, to reduce Cu^II^ to Cu^I^), and FLC were combined in an acid-washed (1 M HCl for ~ 1 h) microcentrifuge tube and allowed to stand for 5 min at room temperature. Plasmid (pBSSK, 0.1 pmol in 130 mmol NaCl) was then added to the reaction mixture and allowed to stand for 5 min at room temperature. H_2_O_2_ (50 μM) was added and allowed to react at room temperature for 30 min. EDTA (50 μM) was added after 30 min to quench the reaction. For the Fe^II^ DNA damage experiments, the indicated FeSO_4_∙7H_2_O concentrations and MES (10 mM, pH 6.0) were used. All concentrations are final concentrations in a 10 μM volume. Samples were loaded into a 1% agarose gel in a TAE running buffer (50×); damaged and undamaged plasmid was separated by electrophoresis (140 V for 60 min). Gels were stained using ethidium bromide and imaged using UV light. The amounts of nicked (damaged) and circular (undamaged) were analyzed using UViProMW (Jencons Scientific Inc., 2007). Intensity of circular plasmid was multiplied by 1.24, due to the lower binding affinity of ethidium bromide to supercoiled plasmid [71]. Statistical significance was determined by calculating p values at 95% confidence (p < 0.05 indicates significance), the Table G listing these values can be found in the Supplementary Information S1 File.

## Supporting information

S1 File Contains Tables A-G and Figures A-I.
(DOCX)
