# Supplementary material for: Fluconazole induces ROS in Cryptococcus neoformans and contributes to DNA damage in vitro
Source: PLoS One. 2018 Dec 7;13(12):e0208471. doi: 10.1371/journal.pone.0208471 (PMC6286144; doi:10.1371/journal.pone.0208471)
Supplement: S1 File — Contains Tables A-G and Figures A-I. (DOCX) [file pone.0208471.s001.docx]

**Fluconazole induces ROS in *Cryptococcus neoformans* and contributes to DNA damage *in vitro***

Congyue Annie Peng, Andrea A. E. Gaertner, Sarah Ana Henriquez, Diana Fang, Rodney J Colon-Reyes, Julia L. Brumaghim, Lukasz Kozubowski

Tables A-G; Figures A-I

**Table A. Oligonucleotides used for reverse transcription and qPCR**

| Name | Sequence |
| --- | --- |
| Oligo(dT)18 | 5’ - TTTTTTTTTTTTTTTTTT - 3’ |
| Cn *MT1* F | 5’- TACGAGCGAGGCTCAAGACA-3’ |
| Cn *MT1* R | 5’- GGCAGCGCCAGATCCGCTG-3’ |
| Cn *MT2* F | 5’ - TTCAACCCCAACCCAGAAAAG-3’ |
| Cn *MT2* R | 5’ - CACCTTGGCAACCGCATGTG-3’ |
| Cn *ACT2* F | 5’ - AAAGCTGGTTTTGCTGGAGA - 3’ |
| Cn *ACT2* R | 5’ - CCGGTTTTGTCTAGGGTTGA - 3’ |
| Cn *SOD1* F | 5’ - CCACCAAATACGCCTCAACT - 3’ |
| Cn *SOD1* R | 5’- GAGGTACAGCCGTTGGTGTT - 3’ |
| Cn *RAD51* F | 5’ - TGGTGCATATCACGACTGGT - 3’ |
| Cn *RAD51* R | 5’ - CCTGGCATAGGCGATATTGT - 3’ |
| Cn *RDH54* F | 5’ - CAAACGCGTTCAGAGAAACA - 3’ |
| Cn *RDH54* R | 5’ - GCACTGACAAATGTGGGATG - 3’ |
| Cn *RAD54* F | 5’ - CTGCCTGTCAAATACGAGCA - 3’ |
| Cn *RAD54* R | 5’ - ATTGGCAGTTAACCGTCCTG - 3’ |
| Cn *SCC1* F | 5’ - GAATACGGTCGTGAGGTCGT - 3’ |
| Cn *SCC1* R | 5’- GTCCCAGATCCATAGGCTCA - 3’ |
| Cn catalase F | 5’ - AGCCGCCAATATGGTTACAG - 3’ |
| Cn catalase R | 5’ - CTGGTGTCCGTCAAAACCTT - 3’ |
| Cn peroxiredoxin F | 5’ - CCATGGACTTCACCTTCGTT - 3’ |
| Cn peroxiredoxin R | 5’ - GTGCTCGTCTGTGAACTGGA - 3’ |
| Cn *TRR1* F | 5’ - CAAGGTTACCGTCCTTTGGA - 3’ |
| Cn *TRR1* R | 5’ - GTCTGGAGGCTCTCGTCATC - 3’ |
| Cn *GAPDH* F | 5’ - TTCTCAGGAACGCCATCGAG - 3’ |
| Cn *GAPDH* R | 5’ CCTTAGCAGCACCAGTGGAA - 3’ |

**Table B. MALDI mass spectrometry data for FLC with Fe^II^ and Cu^II^**

| Metal | m/z (DA) | Metal: ligand |
| --- | --- | --- |
| Copper | 693.1 | 1:2 |
| Iron | 666.7 | 1:2 |

**Table C. CV data for FLC with Fe^II^ and Cu^II^**

| Metal | Epa (mV) | Epc (mV) | ΔE (mV) | E1/2 (mV) |
| --- | --- | --- | --- | --- |
| FLC | - | - | - | - |
| Copper | -83^a^, 121^b^ | -796^a^, -100^b^ | 713^a^, 212^b^ | -440^a^, 11^b^ |
| Copper: FLC |  |  |  |  |
| 1:2 | -73^a^, 342^b^ | -124^a^, 157^b^ | 51^a^, 499^b^ | -99^a^, 250^b^ |
| Iron | 263 | -133 | 396 | 65 |
| Iron: FLC |  |  |  |  |
| 1:2 | 324 | -128 | 452 | 98 |

^a^ Cu^I/0^ potential. ^b^ Cu^II/I^ potential.

**Table D. Gel electrophoresis results for FLC DNA damage assays with Cu^2+^, and 50 µM H_2_O_2_^a^**

| Gel lane | [FLC], µM | % Supercoiled | % Nicked | % Damage Inhibition | *p* Value |
| --- | --- | --- | --- | --- | --- |
| 1: plasmid DNA (p) | 0 | 99.87 ± 0.19 | 0.13 | - | - |
| 2: p + H_2_O_2_ (50 µM) | 0 | 100 ± 0 | 0 | - | - |
| 3: p + FLC + H_2_O_2_ | 25 | 100 ± 0 | 0 | - | - |
| 4: p + FLC | 25 | 100 ± 0 | 0 | - | - |
| 5: p + Cu^+^ (6 µM) + AA (7.5 µM) + H_2_O_2_ | 0 | 4.42 ± 0.05 | 95.58 | - | - |
| 6: p + Cu^2+^ (12.5 µM )+ H_2_O_2_ | 0 | 100 ±0 | 0 | -1 ± 0 | < 0.001 |
| 7: p + Cu^2+^ (0.05 μM) + FLC + H_2_O_2_ | 0.5 | 99.91 ± 0.12 | 0.09 | -0.91 ± 0 | < 0.001 |
| 8: p + Cu^2+^ (0.5 μM) + FLC + H_2_O_2_ | 1 | 100 ± 0 | 0 | -1 ± 0 | < 0.001 |
| 9: p + Cu^2+^ (2.5 μM) + FLC + H_2_O_2_ | 5 | 100 ± 0 | 0 | -1 ± 0 | < 0.001 |
| 10: p + Cu^2+^ (5 μM) + FLC + H_2_O_2_ | 10 | 96.68 ± 2.54 | 3.32 | 2.32 ± 2.54 | 0.254 |
| 11: p + Cu^2+^ (9 μM) + FLC + H_2_O_2_ | 18 | 81.57 ± 2.49 | 18.43 | 17.43 ±2.49 | 0.007 |
| 12: p + Cu^2+^ (12.5 μM) + FLC + H_2_O_2_ | 25 | 63.33 ± 5.6 | 36.67 | 35.67 ± 5.6 | 0.0081 |

^a^Data are reported as the average of three trials with calculated standard deviations shown.

**Table E. Gel electrophoresis results for FLC DNA damage assays with Fe^2+^, and 50 µM H_2_O_2_^a^**

| Gel lane | [FLC], µM | % Supercoiled | % Nicked | % Damage | *p* Value |
| --- | --- | --- | --- | --- | --- |
| 1: plasmid DNA (p) | 0 | 100 ±0 | 0 | - | - |
| 2: p + H_2_O_2_ (50 µM) | 0 | 100 ± 0 | 0 | - | - |
| 3: p + FLC + H_2_O_2_ | 50 | 100 ± 0 | 0 | - | - |
| 4: p + FLC | 50 | 100 ± 0 | 0 | - | - |
| 5: p + Fe^2+^ (2 μM) + H_2_O_2_ | 0 | 6.35 ±5,53 | 93.65 | - | - |
| 6: p + Fe^2+^ (0.005 μM) + FLC + H_2_O_2_ | 0.01 | 100 ± 0 | 0 | -1 ± 0 | < 0.001 |
| 7: p + Fe^2+^ (0.05 μM) + FLC + H_2_O_2_ | 0.1 | 90.20 ± 0.10 | 9.8 | 8.8 ± 0.01 | < 0.001 |
| 8: p + Fe^2+^ (0.25 μM) + FLC + H_2_O_2_ | 0.5 | 60.46 ± 0.58 | 39.54 | 38.54 ± 0.58 | < 0.001 |
| 9: p + Fe^2+^ (0.5 μM) + FLC + H_2_O_2_ | 1 | 61.65 ±1.22 | 38.35 | 37.35 ± 1.22 | < 0.001 |
| 10: p + Fe^2+^ (1 μM) + FLC + H_2_O_2_ | 2 | 11.95 ± 5.69 | 88.05 | 87.05 ± 5.69 | 0.001 |
| 11: p + Fe^2+^ (2.5 μM) + FLC + H_2_O_2_ | 5 | 3.03 ± 4.28 | 96.97 | 95.97 ± 4.28 | < 0.001 |
| 12: p + Fe^2+^ (5 μM) + FLC + H_2_O_2_ | 10 | 3.79 ± 0.76 | 96.21 | 95.21 ± 0.76 | < 0.001 |
| 13: p + Fe^2+^ (25 μM) + FLC + H_2_O_2_ | 50 | 4.43 ± 2.02 | 95.57 | 94.57 ± 2.02 | < 0.001 |

^a^Data are reported as the average of three trials with calculated standard deviations shown.

**Table F. Gel electrophoresis results for Fe^2+^ DNA damage assays with 50 µM H_2_O_2_^a^**

| Gel lane | [Fe^2+^], µM | % Supercoiled | % Nicked | % Damage Inhibition | *p* Value |
| --- | --- | --- | --- | --- | --- |
| 1: plasmid DNA (p) | 0 | 100 ± 0 | 0 | - | - |
| 2: p + H_2_O_2_ (50 µM) | 0 | 100 ± 0 | 0 | - | - |
| 3: p + Fe^2+^ + H_2_O_2_ | 0.005 | 100 ± 0 | 0 | -1 ± 0 | < 0.001 |
| 4: p + Fe^2+^ + H_2_O_2_ | 0.05 | 100 ± 0 | 0 | -1 ± 0 | < 0.001 |
| 5: p + Fe^2+^ + H_2_O_2_ | 0.25 | 100 ± 0 | 0 | -1 ± 0 | < 0.001 |
| 6: p + Fe^2+^ + H_2_O_2_ | 0.5 | 100 ± 0 | 0 | -1 ± 0 | < 0.001 |
| 7: p + Fe^2+^ + H_2_O_2_ | 1 | 53.49 ± 5.0 | 46.51 | 45.51 ± 5.0 | 0.004 |
| 8: p + Fe^2+^ + H_2_O_2_ | 2 | 9.51 ± 5.88 | 90.49 | 89.49 ± 5.88 | 0.001 |
| 9: p + Fe^2+^ + H_2_O_2_ | 5 | 0.73 ± 1.03 | 99.27 | 98.27 ± 1.03 | < 0.001 |
| 10: p + Fe^2+^ + H_2_O_2_ | 12.5 | 1.51 ± 2.14 | 98.49 | 97.49 ± 2.14 | < 0.001 |

^a^Data are reported as the average of three trials with calculated standard deviations shown.

**Table G. Statistical analysis of Fe^2+^ and Fe^2+^ FLC DNA damage through one-tailed T-Test analysis**

| [Fe^2+^], µM | % DNA Damage Fe^2+^ | % DNA Damage Fe^2+^ + FLC | *p* Value |
| --- | --- | --- | --- |
| 0.005 | -1 ± 0 | -1 ± 0 | < 0.001 |
| 0.05 | -1 ± 0 | 8.8 ± 0.01 | < 0.001 |
| 0.25 | -1 ± 0 | 38.54 ± 0.58 | < 0.001 |
| 0.5 | -1 ± 0 | 37.35 ± 1.22 | < 0.001 |
| 1 | 45.51 ± 5.00 | 87.05 ± 5.69 | 0.006 |
| 2 | 89.49 ± 1.03 | 95.97 ± 4.28 | 0.120 |
| 5 | 98.27 ± 2.14 | 95.21 ± 0.76 | 0.002 |

**
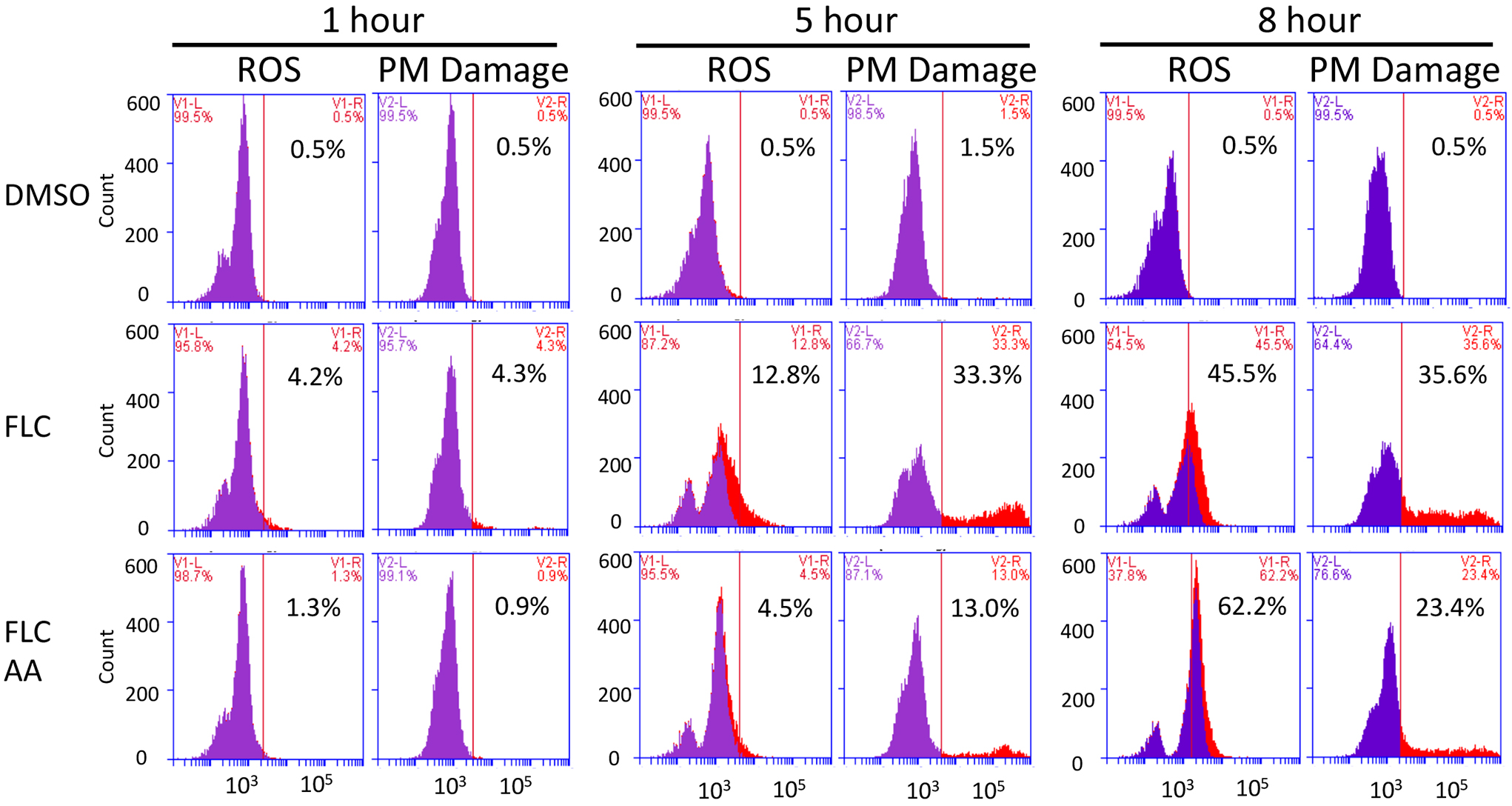
**

**Figure A**

**FLC effects on ROS and plasma membrane damage**

Cells (strain H99) were incubated in YPD medium supplemented with 0.1% (v/v) DMSO, 32 µg/ml or 32 µg/ml with 10 mM ascorbic acid at 24°C for 1, 5 and 8 hours. ROS (fluorescence of the H_2_DCFDA) and plasma membrane (PM) damage (fluorescence of the propidium iodide (PI)) was detected by flow cytometry. Vertical lines in the graphs indicate arbitrary boundary between background and elevated levels of the H_2_DCFDA and PI fluorescence whereas percentages indicate fraction of cells with elevated fluorescence. In the ROS graphs, purple area indicates the ROS content in the cell subpopulation that shows background PI fluorescence. Orange indicates ROS content in the cell subpopulation with elevated PI stain.

**
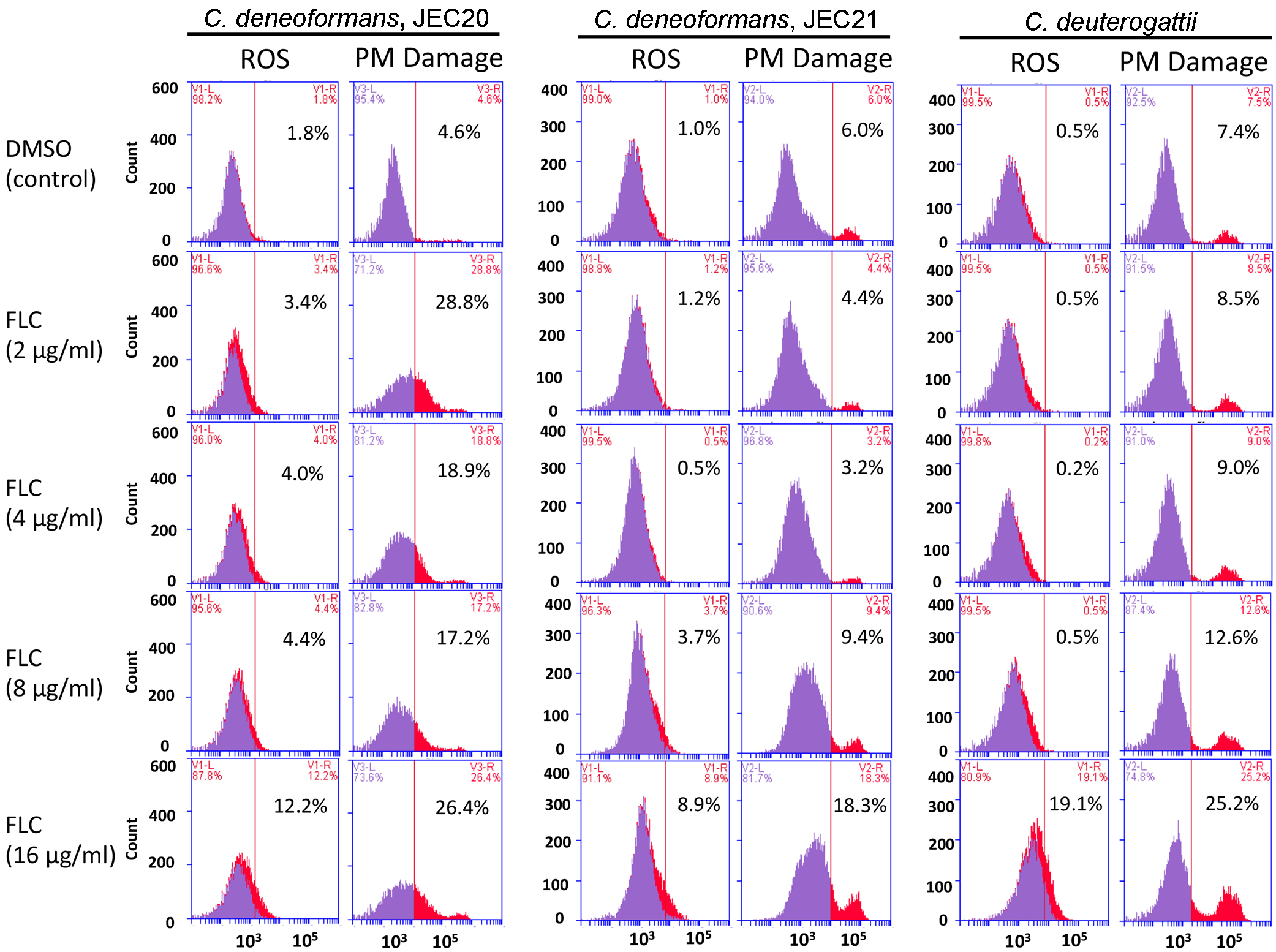
**

**Figure B**

**The effect of FLC on ROS and plasma membrane integrity in *C. neoformans* var. *neoformans* (*C. deneoformans*: JEC20, JEC21) and *C. deuterogattii* (R265)**

Cells were incubated in YPD medium supplemented with 0.1% (v/v) DMSO (control) or indicated concentrations of FLC for 8 hours at 24°C. ROS (H_2_DCFDA) and plasma membrane damage (propidium iodide (PI)) was detected by flow cytometry. Vertical lines in the graphs indicate arbitrary boundary between background and elevated levels of H_2_DCFDA and PI fluorescence whereas percentages on the right indicate fraction of cells with elevated fluorescence. In the ROS graphs, purple area indicates the ROS content in the cell subpopulation that shows background PI fluorescence. Orange indicates ROS content in the cell subpopulation with elevated PI.

| 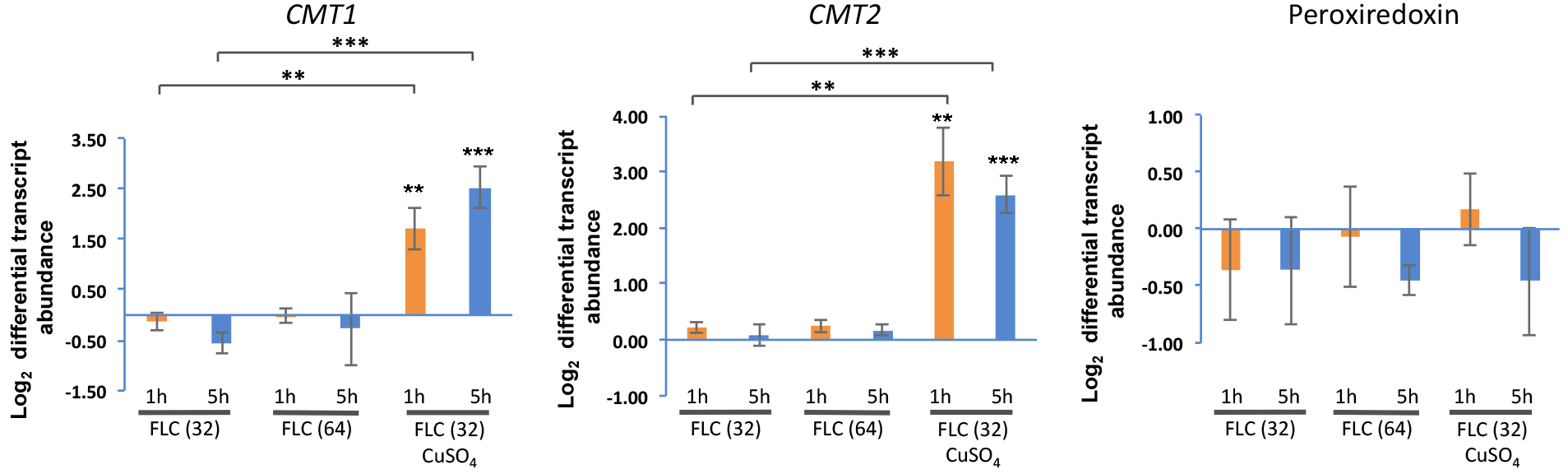  **Figure C**  **The effects of FLC on transcription of *C. neoformans* *CMT1*, *CMT2*, and peroxiredoxin**  Exponentially grown cultures of *C. neoformans* (H99) were resuspended in YPD medium supplemented with FLC (32 or 64 µg/ml), FLC (32 µg/ml) and CuSO_4_ (1 mM), or 0.1% (v/v) DMSO (as control) and grown at 24°C for 1 and 5 hours prior to total RNA extraction. Transcript abundance was analyzed using quantitative PCR. Statistical significance is indicated as double (p < 0.01), or triple star (p < 0.001). |
| --- |
| 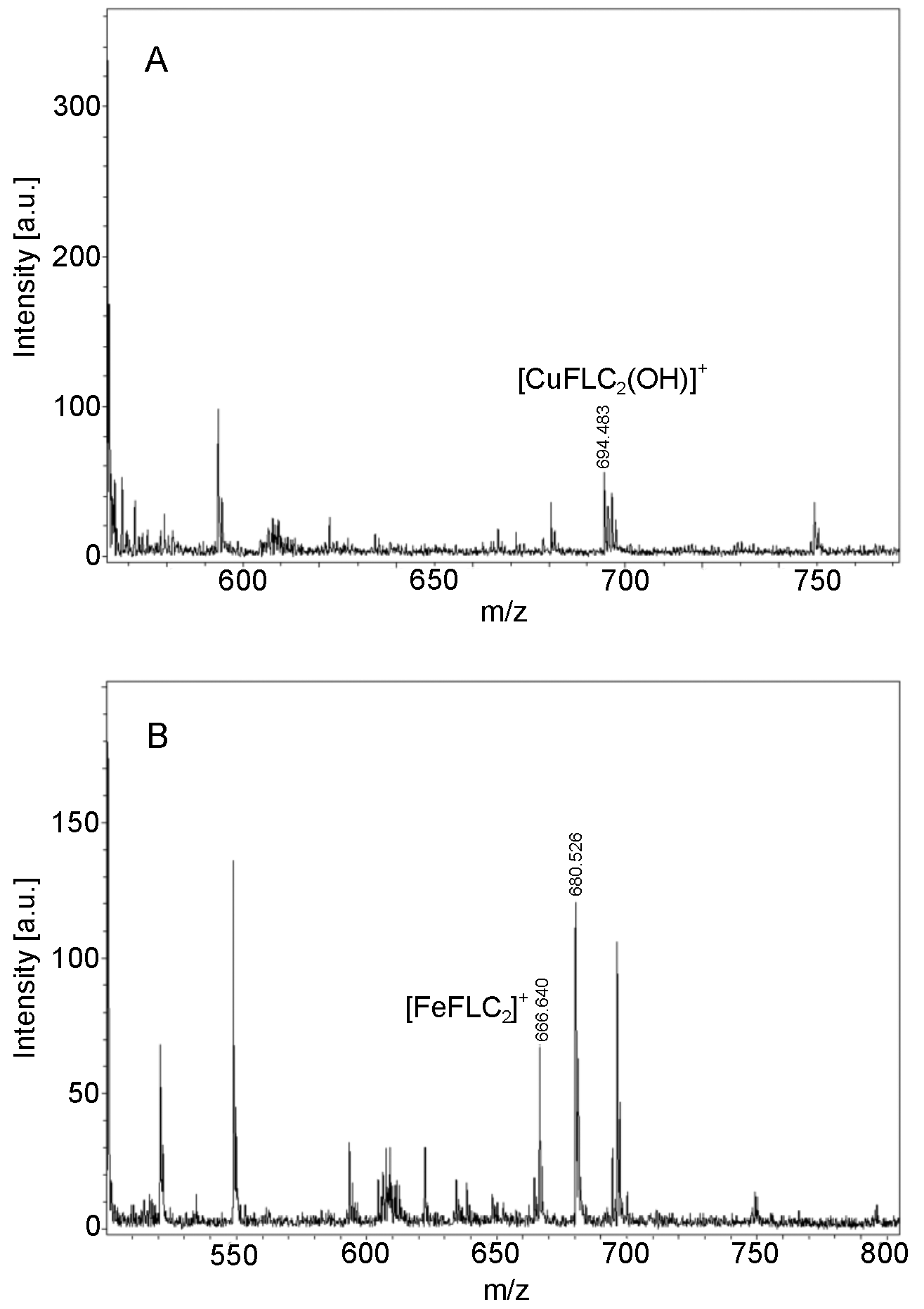 |

**Figure D**

**MALDI-TOF MS spectra of the FLC-metal species A) [CuFLC_2_(OH)]^+^ and B) [FeFLC_2_-H^+^]^+^**


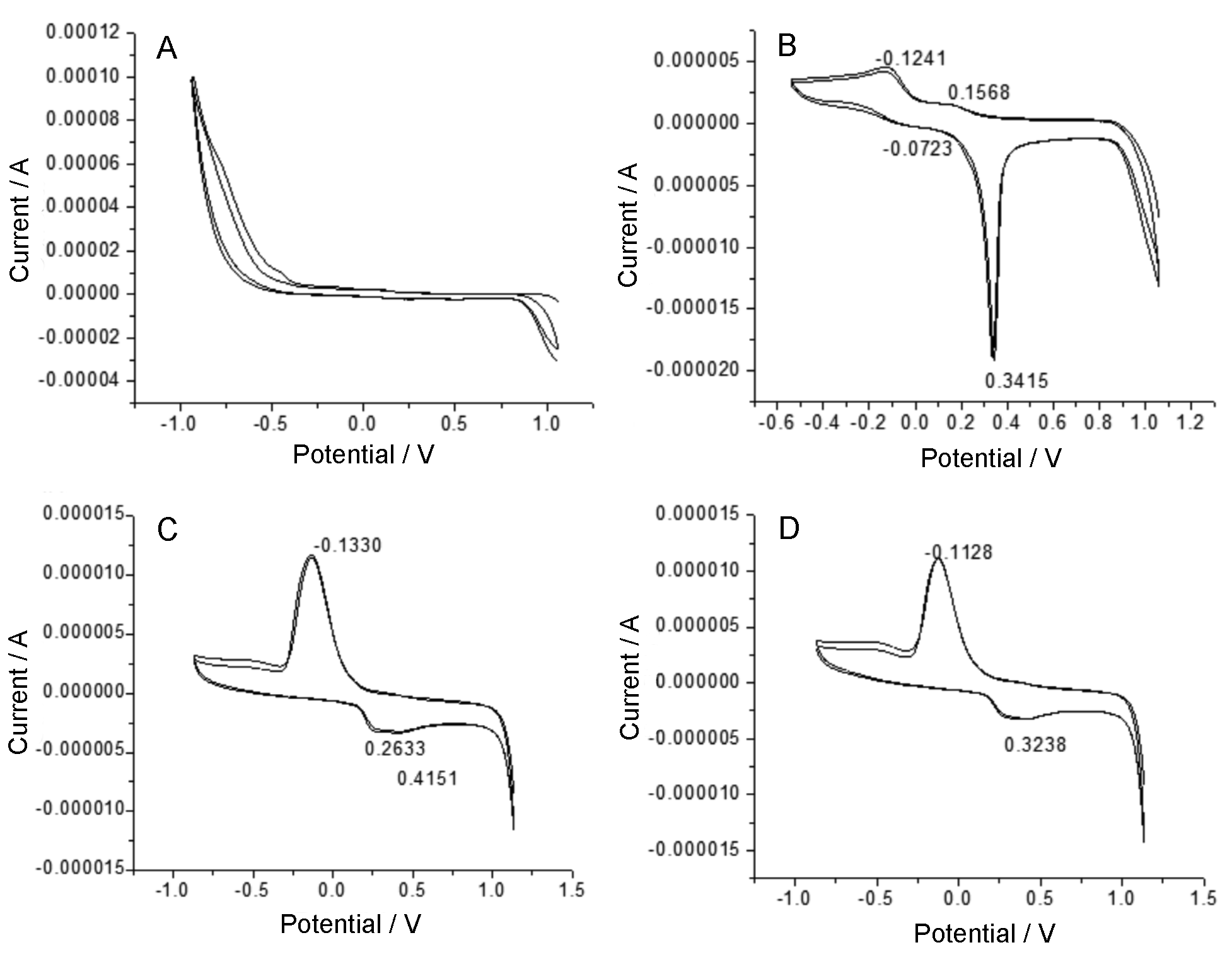


|  |  |
| --- | --- |
|  |  |

**Figure E**

**Cyclic voltammograms for A) CuSO_4_ (100 µM); B) 1:2 Cu^II^:FLC (100:200 µM) in MOPS buffer (10 mM, pH 7.0); C) FeSO_4_ (300 µM); and D) 1:2 Fe^II^:FLC (300:600 µM) in MES buffer (10 mM, pH 6.0).**

All contain KNO_3_ (10 mM) as a supporting electrolyte. All solutions were either cycled between -1.0 and 1.0 V for the iron experiments and -0.60 and 1.0 V for the copper experiments. Potentials are given vs. NHE.


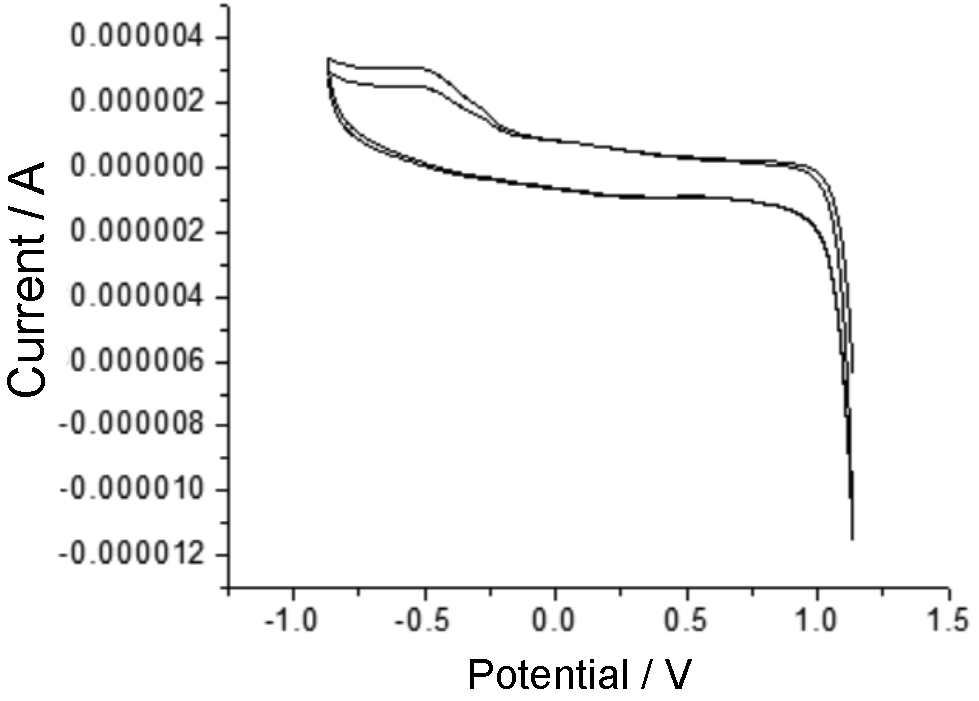


**Figure F**

**Cyclic voltammograms for FLC (300 μM) in MES buffer (10 mM, pH =6.0) with KNO_3_ as a supporting electrolyte.**

The solution was cycled between -1000 mV and 1000 mV vs. NHE at a scan rate of 100mV/s.


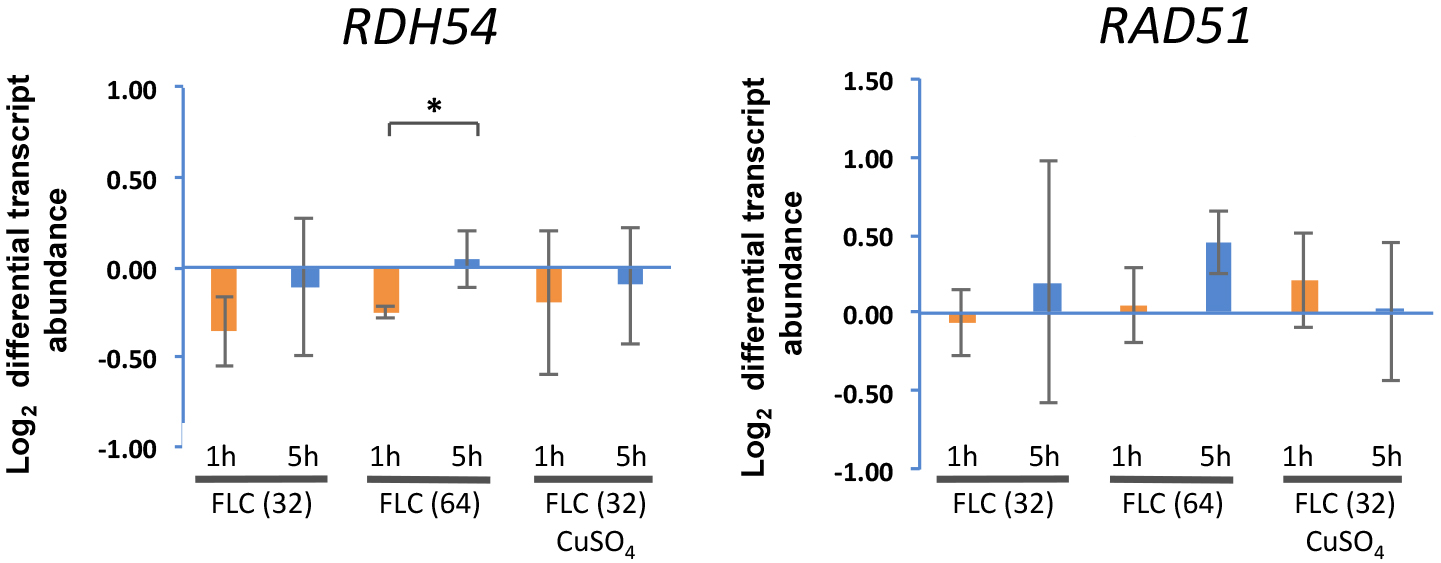


**Figure G**

**The effects of FLC on transcription of *C. neoformans RDH54* and *RAD51***

Exponentially grown cultures of *C. neoformans* (H99) were resuspended in YPD medium supplemented with FLC (32 or 64 µg/ml), or FLC (32 μg/ml) and CuSO4 (1 mM), or 0.1% (v/v) DMSO (as control) and grown at 24°C for 1 and 5 hours prior to total RNA extraction. Transcript abundance was analyzed using quantitative PCR. Star indicates statistical significance (p< 0.05).

**
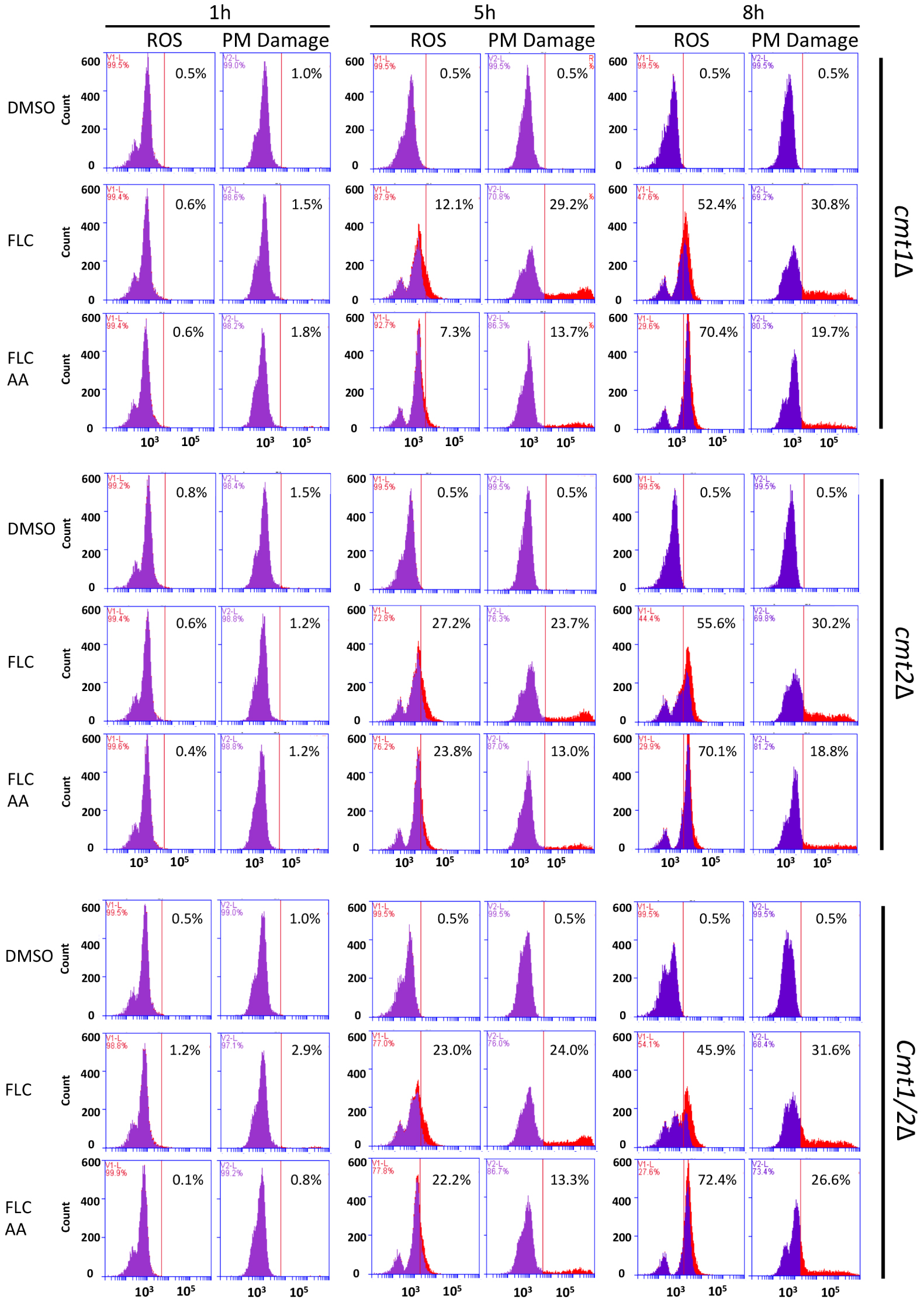
**

**Figure H. The effect of FLC on ROS and plasma membrane damage and the effect of ascorbic acid on ROS and plasma membrane damage on FLC-treated MT mutant strains**

Cells (*cmt1*Δ, *cmt2*Δ and *cmt1/2*Δ) were incubated in YPD medium supplemented with 0.1% (v/v) DMSO (control), 32 µg/ml FLC or FLC+10 mM ascorbic acid (AA) at 24°C for 1, 5, or 8 hours. ROS (fluorescence of the H_2_DCFDA) and plasma membrane (PM) damage (fluorescence of the propidium iodide (PI)) was detected by flow cytometry. Vertical lines in the graphs indicate arbitrary boundary between background and elevated levels of the H_2_DCFDA and PI fluorescence whereas percentages indicate fraction of cells with elevated fluorescence. In the ROS graphs, purple area indicates the ROS content in the cell subpopulation that shows background PI fluorescence. Orange indicates ROS content in the cell subpopulation with elevated PI stain.

**
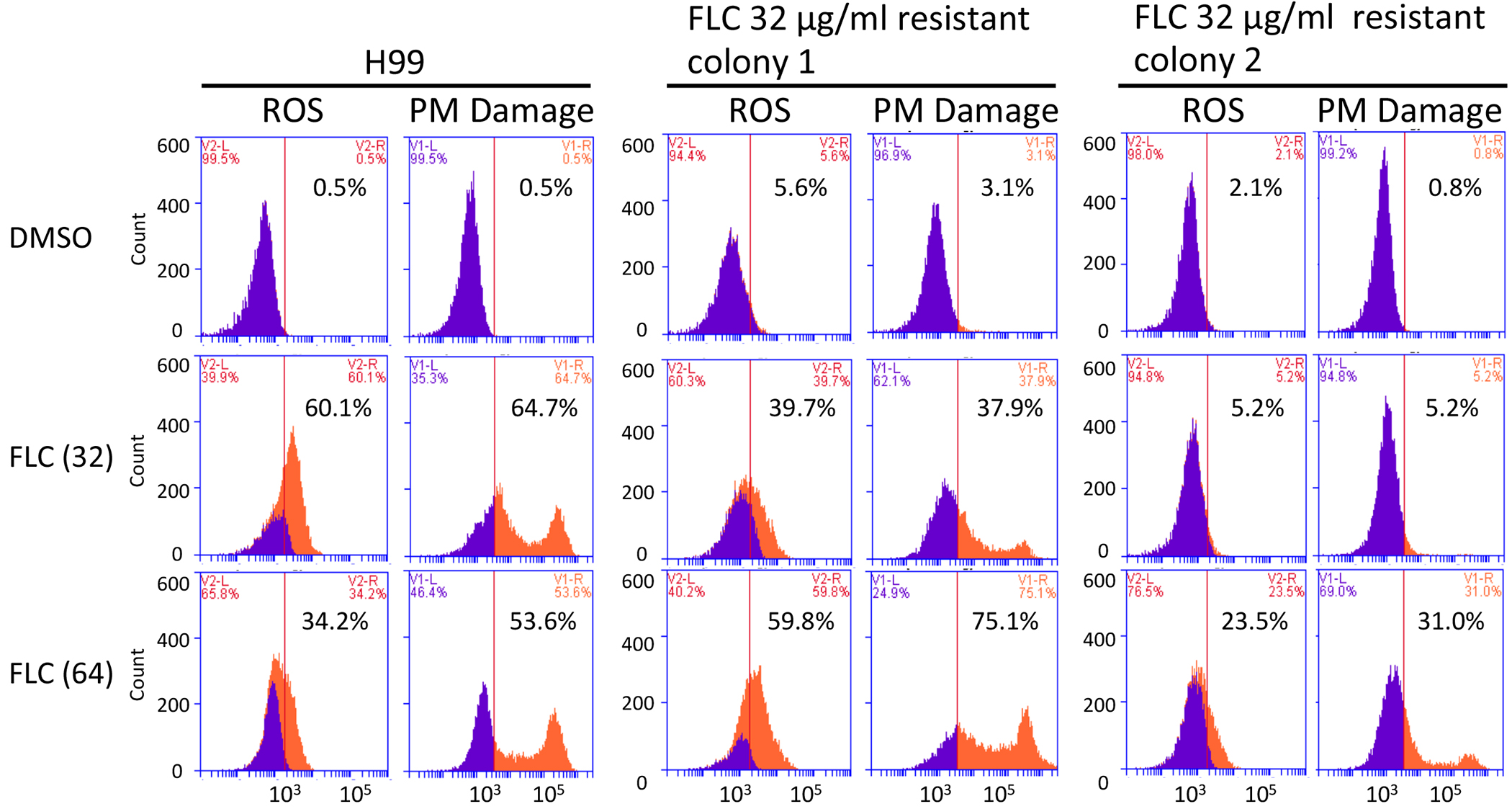
**

**Figure I**

**Differential FLC effects on ROS and plasma membrane damage on cells from two colonies selected from the FLC resistant *C. neoformans* cells.**

Cells (strain H99) were incubated in YPD with FLC 32 μg/ml medium for 4-5 days. Colonies with bigger size were picked and inoculated on a fresh YPD with FLC 32 μg/ml plate. Two colonies were randomly picked from the plate and cultured in YPD liquid medium supplemented with 0.1% (v/v) DMSO, 32, 64µg/ml FLC at 24°C for 24h. ROS (fluorescence of the H_2_DCFDA) and plasma membrane (PM) damage (fluorescence of the propidium iodide (PI)) was detected by either flow cytometry. Vertical lines in the graphs indicate arbitrary boundary between background and elevated levels of the H_2_DCFDA and PI fluorescence whereas percentages indicate fraction of cells with elevated fluorescence. In the ROS graphs, purple area indicates the ROS content in the cell subpopulation that shows background PI fluorescence. Orange indicates ROS content in the cell subpopulation with elevated PI stain.
